# Voluntary isometric contractions at maximal shortening as a new technique to achieve neuromuscular re-education in healthy subjects

**DOI:** 10.1016/j.jesf.2024.12.003

**Published:** 2024-12-18

**Authors:** Julio-Artemi Hernández-Trujillo, María-Dolores González-Rivera, Jorge M. González-Hernández

**Affiliations:** a Universidad de Alcalá, Facultad de Medicina y Ciencias de la Salud, Departamento de Ciencias Biomédicas, Alcalá de Henares, Madrid, Spain; b BioReed Lab, Tenerife, Canarias, Spain; c Faculty of Health Sciences, EVOPRED Research Group, Universidad Europea de Canarias, La Orotava, Tenerife, Spain

**Keywords:** Ballistic stretching, Isometric training, Maximal strength, Neuromuscular re-education, Shortened muscle position

## Abstract

**Background:**

*objectives*: Neuromuscular re-education has focused on improving motor activities in patients with pathologies by retraining the nervous system. However, this has not yet been investigated in healthy individuals. Voluntary isometric contractions at maximal muscle shortening (VICAMS) is a new technique with the same objective. This study aimed to investigate the chronic effects of these techniques on range of motion, strength, and vertical jump.

**Methods:**

Sixty healthy, recreationally active participants (mean age: 46.4 ± 5.5), were randomly assigned to three groups (VICAMS, ballistic stretching, and control) who were trained for eight weeks. To assess chronic effects, active range of motion, maximal isometric strength, and countermovement jump height were determined before and after the intervention.

**Results:**

Main effects of time and time∗group interactions were found for all variables (p < 0.001). Between-group differences were observed in the VICAMS group after the intervention, with significantly higher flexibility and strength values compared to the other groups. Intra-group differences were observed in the VICAMS and ballistic groups, as the values for all variables increased from baseline. In the VICAMS group, increases were observed in both flexibility (19.15 %) and strength (47.63 %). Increases in flexibility (2.59 %) and strength (1.84 %) were also observed in the ballistic group. For jumping, intra-group differences showed that both the VICAMS (16.56 %) and ballistic (4.34 %) groups had improved values compared to baseline values.

**Conclusion:**

Our findings suggest that VICAMS is an effective, simple, and inexpensive alternative to conventional training methods for improving flexibility and strength in rehabilitation.

## 1 Introduction

Neuromuscular re-education (NR) is concerned with retraining the brain and spinal cord in motor activities using physiological principles of the muscular and nervous systems.^1^ These principles were first described by Kabat & Knott^2^ in patients with spastic paralysis. Since then, research has primarily focused on the clinical treatment of pathologies.^3^, ^4^, ^5^ However, there is no consensus on the methodology to be followed, which has led to using different techniques to achieve the same goal.^6^ Currently, there is no evidence in the literature regarding the effects of these techniques on healthy individuals.

Although the principles of NR focus on the treatment of patients with pathologies, they could be applied to improve motor function in other sectors of the population, such as recreational physical activity or sport. Repeated activation of brain-muscle communication is essential for the development of voluntary movement.^7^ The ability of the nervous system to appropriately activate muscles determines the ability to develop strength.^8^ Likewise, active and passive flexibility and muscular endurance improve as repetitions are performed.^2^ Training based on voluntary isometric contractions against resistance^1^ is an effective method to improve strength.^9^ Muscle length at the moment of contraction is one of the main variables that influence isometric training.^10^ Thus, in contractions in which the muscle is shortened, neural activation is greater,^11^ and strength gains are especially due to neural adaptations.^12^ Furthermore, when the muscle is shortened, gamma motor neurons must stretch the intrafusal fibers so that the neuromuscular spindles can send appropriate information to the central nervous system and prevent injury.^13^ To date, only a few studies have investigated the chronic effect of isometric training on short muscle lengths^10^^,^^14^ but not on maximal shortened muscle positions.

In addition to isometric training, maximum muscle shortening can be achieved in sports activities through ballistic stretching (BS), which is often used by athletes and is characterized by actively reaching the maximum joint range.^15^ It should be noted that there is a lack of consensus in the literature to describe the procedures for performing BS. Several authors have included them alongside dynamic stretching in reviews of their effects on performance.^16^^,^^17^ Dynamic stretches are controlled movements through the active range of motion of each joint, which can be executed slowly or quickly.^18^ Whereas BS involves accelerating a mass at high speed throughout the movement.^19^ Owing to their similar characteristics, dynamic stretching performed at high speed was included alongside the BS in this study.

Our research group applied voluntary isometric contractions at maximal shortening (VICAMS) and found acute improvements in active flexibility, maximal isometric strength, and vertical jumps.^20^ In VICAMS, the researcher must bring the joint to the maximal possible muscle shortening and then exert force in the opposite direction, while the participant must try to resist this force. However, the chronic effects of isometric training on maximal muscle shortening have not yet been studied. Similarly, there are few studies on the chronic effects of BS on performance. Therefore, the main aim of this study was to investigate the effect of VICAMS application as an NR technique on the performance of physically active individuals after eight weeks of training. It was hypothesized that VICAMS would provide better results than BS in the variables of active range of motion (AROM), maximal voluntary isometric strength (MVIF), and countermovement jump height (CMJ).

## 2 Material and methods

### 2.1 Study design

Participants were randomly divided into three groups: the BS group, VICAMS group, and control group (CG). A randomized controlled repeated-measures design was used to determine possible differences in changes in AROM, MVIF, and CMJ after BS and VICAMS. Participants underwent two weeks of familiarization with the tests before the measurements were taken. Males and females were equally distributed across all groups. AROM, MVIF, and CMJ values were determined before the first intervention and after eight weeks to assess the chronic effects. AROM and MVIF were measured for hip extension (HE) and knee extension (KE). The CG was measured again at eight weeks, but did not undergo any intervention.

### 2.2 Participants

A total of 60 healthy, recreationally active participants^21^ volunteered for the present study. Each group consisted of 20 participants: BS group (10 females and 10 males; age 46.7 ± 5.9 years; height 1.72 ± 0.08 m; weight 76.9 ± 8.7 kg; body mass index (BMI) 25.9 ± 1.7 kg/m^2^); VICAMS group (10 females and 10 males; age 44.7 ± 5.1 years; height 1.70 ± 0.05 m; weight 72.8 ± 8.0 kg; body mass index (BMI) 25.3 ± 2.4 kg/m^2^); and CG (10 females and 10 males; age 47.9 ± 5.1 years; height 1.71 ± 0.05 m; weight 74.1 ± 8.2 kg; body mass index (BMI) 25.4 ± 2.9 kg/m^2^). This study conformed to CONSORT guidelines.^22^ Inclusion criteria were as follows: (1) age 35–55 years; (2) non-competitive physical activity at least twice a week. Participants with joint or muscle diseases that prevented correct performance of the tests were excluded from the study. The participants were informed that they should not engage in physical activity the day before the test. During the familiarization sessions, the study design was explained to the athletes, and they were allowed to ask questions. All participants provided written informed consent. The study was approved by the Ethical Committee for Animal Research and Experimentation of the University of Alcalá (CEID/2022/4/083) and was conducted in accordance with the principles established in the Declaration of Helsinki.

### 2.3 Measurements

**Fig. 1:**
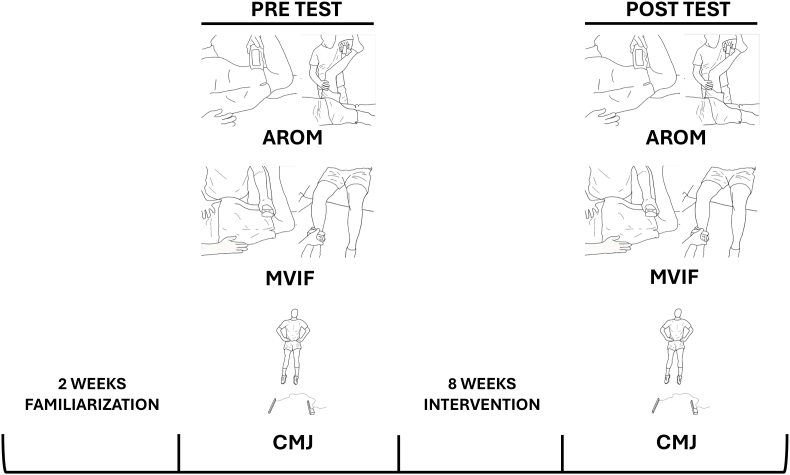
Scheme of study design. The order in which the study was carried out and the variables that were measured are shown: AROM (Active Range of Motion), MVIF (Maximal Voluntary Isometric Force, CMJ (Counter Movement Jump).

To ensure the high reliability of the study, all measurements were carried out by the same researcher with extensive experience in musculoskeletal assessment. The measuring devices used are reliable when used by the same examiner.^23^ The laboratory was maintained at a constant temperature of approximately 23 °C. Before the measurements, the participants were required to complete a standardized warm-up of the hip and knee, first with slow movements, and then in a ballistic manner. The participants were instructed to reach the maximum possible joint range for each repetition. The warm-up consisted of five flexions of each hip flexing the knee, five abductions of each hip, five extensions of each hip, five flexions of each knee, and 10 heel raises at a time. Finally, 5 squats and 3 CMJs were performed. The measurements were then performed in the following order: (1) AROM (3-min pause), (2) MVIF (3-min pause), and (3) CMJ. The intervention was initiated immediately after the initial measurements. To avoid acute effects, the next measurements were performed three days after the last intervention. The same protocol was used. A schematic representation of the study design is shown in Fig. 1.

#### 2.3.1 Active range of motion (AROM)

AROM measurements of the hip and knee were performed according to the method described by Norkin and White.^24^ Thus, HE was measured in the prone position and KE in the supine position in the same order to ensure the reliability of the process. A calibrated hand-held digital goniometer-dynamometer model Micro-FET3 (Hoggan Scientific, Salt Lake City, Utah, USA) was used to measure AROM and MVIF, which provides reliability and ease of measurement.^25^ Anatomical position was recorded using a goniometer as the starting position. Subsequently, the participant was instructed to perform a slow, controlled movement to the maximum possible joint amplitude, where the measurement was recorded. Three measurements were taken for each joint position, and the highest value was used for the subsequent data analysis.^26^ The researcher ensured that no compensatory movements had occurred. A rest period of 10 s was allowed between measurements to minimize the effect of fatigue, and 30 s was allowed between positions to fix the participant's posture.

#### 2.3.2 Maximal voluntary isometric force (MVIF)

Hip strength was measured using the positions and techniques described by Thorborg^27^ and knee strength was measured following the method described by Andrews.^28^ Thus, HE was measured in the prone position and the KE in the sitting position. Force measurements were expressed in newtons and measurement values were provided by our scientifically validated device (Microfet 3, Hoggan Scientific, Salt Lake City, Utah, USA).^23^ As in the previous protocol, three measurements were taken, and the highest was used for the analysis.^29^ For its execution, participants were instructed to exert as much force as possible against the device, while the researcher held it as still as possible without breaking the position. This methodology is known as the ‘make test’ and is commonly used in manual dynamometry because of its reliability.^30^ Participants were asked to gradually increase to maximal force and maintain the effort for a total time of 5 s,^27^ which was sufficient to reach maximal force.^31^ The commands were push, hold, and relax. To reduce the fatigue effect, the subjects rested for 15 s between each measurement and 30 s between positions. The device pad and examiner's forearm were held perpendicular to the limb being measured.^28^ When the subject did not demonstrate maximal effort, did not follow instructions, or the HHD recorded an error message, the test was repeated with appropriate rest compensation.

#### 2.3.3 Countermovement jump (CMJ)

During the warm-up, the participants were familiarized with jumping and landing techniques. The Bosco^32^ protocol was used for the evaluation, and jump height was recorded in centimeters. Measurements were performed using a 420 × 590 mm Chronojump contact platform (Spain), which allows reliable and cost-effective measurement of jump height and changes in physical performance.^33^ Three jumps were executed with 1 min rest in between^34^ to avoid the effect of fatigue.^35^ The highest value was selected for analysis.^36^ The execution technique consisted of placing both hands at the hip height, bending the knees to a depth selected by the participant, and attempting to jump as high as possible in a single movement.^37^ As participants were inexperienced in CMJ, knee flexion close to 90 °was suggested.^10^ The participants were instructed to minimize lateral and horizontal displacements during the jumps.^38^ The jump was repeated when the execution was incorrect, which could have affected the measurement. Verbal encouragement was provided to help achieve maximal effort in each jump.^39^

### 2.4 Intervention

#### 2.4.1 Ballistic stretching protocol (BS)

It was performed twice a week for eight weeks (a total of 16 interventions), with at least two days between each intervention. The participants were asked to continue their usual physical activity during this period. Each session consisted of three sets of 20 s on each leg for each exercise, with a recovery of 10 s each time. The total time was approximately 18 min. To achieve similar performance among participants, a tempo of 40 beats per min, measured by a metronome, was set.^40^^,^^41^ They were instructed to follow the metronome beat and complete the maximum possible range of motion without bouncing.^42^ The order and exercises performed were.

1) Hip flexion (HF). The participant stood with hands on hips and was instructed to lift one leg forward by bending the knee and return to the starting position.
2) Hip abduction (HABD). The participant stood with hands resting on the wall and was instructed to spread one leg out to the side with the knee extended and return to the starting position.
3) Hip Adduction (HADD). The participant stood with hands resting on the wall and was instructed to slightly move the supporting leg back and bring the free leg forward just in front. From this position, the free leg performed maximal adduction and returned to the starting position.
4) Hip extension (HE). The participant stood with hands resting on the wall and was instructed to move the leg backwards with the knee extended and return to the starting position.
5) Knee flexion (KF): The participant stood with hands resting on the wall and was instructed to bend the knee and return to the starting position.
6) Knee extension (KE): The participant lay supine with one hip and one knee flexed to 90°, holding the leg with the hands behind the knee to remove the force from the hip flexors. He/she was instructed to extend the knee and return to the starting position.

#### 2.4.2 Voluntary isometric contraction at maximal shortening protocol (VICAMS)

**Fig. 2:**
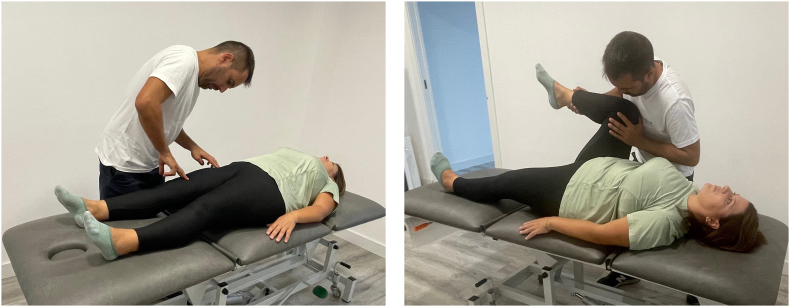
Example of muscle palpation and VICAMS hip flexion.

The duration, frequency, and joint movements selected for this protocol were similar to those selected for the BS protocol. The selection of the exercises was based on the principles of NR,^1^^,^^2^ which indicate the repetition of simple voluntary movements, with maximal isometric activation against the manual resistance of the researcher to activate the greatest number of motor units. To perform them, the researcher brought the joint to the maximal possible muscle shortening and then exerted force in the opposite direction while the participant tried to resist this force. To optimize the participant's muscle contraction, before each exercise, the subject was shown where the main muscles responsible for the movement were located and where they had to concentrate to exert force. In addition, the subject was palpated with two fingers at various points along the path of these muscles to make it easier to locate them.^1^ The participant was asked to resist by maintaining a constant opposing force of similar intensity to the one he/she was receiving. An example of muscle palpation and VICAMS hip flexion is shown in Fig. 2.

The total intervention time was approximately 18 min, similar to that of BS. Each voluntary contraction lasted for 5 s. Nine repetitions of each movement were performed with 3 s of rest between repetitions, 15 s between each leg, and 30 s between exercises. The intensity of the applied force was divided into three levels: three repetitions at light intensity (20–30 %), three repetitions at medium intensity (45–55 %), and three repetitions at maximum intensity (100 %). Individual intensity thresholds were recorded for each subject to work in their zone. After each repetition, the researcher maintained the shortening position and insisted on it until the maximum was reached. To minimize stimuli in other directions, the forearm was kept perpendicular to the segment where the force was applied. Verbal cues were contract, hold, and relax during execution. The order of the exercises was similar to that in the BS protocol. All exercises were performed on a stretcher in the supine position, except for HE and knee flexion and extension, which were performed in the prone position.

### 2.5 Statistical analysis

Statistical analysis was performed using the statistical program SPSS of International Business Machines (IBM), version 21.0 (Chicago, IL, 224 USA). Standard statistical methods were used to calculate mean values and standard deviations (SD). Beforehand, the Kolmogorov-Smirnov test was performed to examine the normality of the data distribution, and the data were tested for normal distribution. The sample size was calculated using GRANMO version 7.12 (Barcelona, Spain). Accepting a significance level of 5 % and statistical power of 80 %, 14 participants were required for each group. The standard deviation considered was 6.4 °based on previous studies.^43^ Independent samples Student's t-tests was used to compare the baseline characteristics of the participants. As there were no differences between groups at baseline, a two-way repeated measures analysis of variance (ANOVA) (group × time) with Bonferroni post hoc test was used to assess group-by-time interactions and within-group and between-group effects. We obtained 95 % confidence intervals (CI) for all differences and effect sizes (ES) in case of significant differences, interpreted using Cohen's d, considering small (0.1), moderate (0.3), large (0.5), very large (0.7) and extremely large (0.9) effects.^44^ The significance level for all statistical tests was set at P < 0.05.

## 3 Results

Table 1 shows all data for the variables AROM, MVIF, and CMJ for all participants according to the group to which they belonged. Main time effects and time × group interactions were found for all variables (p < 0.001).

**Table 1:** Data of active range of motion (hip and knee), maximal voluntary isometric force (hip and knee) and countermovement jump for pre and post measurements in participants belonging to all groups.

		Pre		Post		Intra-group differences (pre vs post)			Between-group differences (at post)			
	Group	Mean	SD	Mean	SD	Mean	95%CI	ES (d)		Mean	95%CI	ES (d)
AROM Right Hip Extension (degrees)	Ballistic	17.65	5.52	18.50	6.30	0.85	(0.28–1.42)	0.14	Ballistic vs Vicams	5.00	(0.41–9.59)	0.82
	Vicams	18.40	5.22	23.50	5.88	5.10	(4.50–5.71)	0.92	Vicams vs control	6.60	(2.01–11.19)	1.17
	Control	17.5	5.40	16.90	5.43	0.15	(-0.50-0.20)	NS	Control vs ballistic	1.60	(-2.99-6.19)	NS
AROM Left Hip Extension (degrees)	Ballistic	17.85	5.73	19.00	6.12	1.15	(0.54–1.76)	0.19	Ballistic vs Vicams	7.15	(2.08–12.22)	1.03
	Vicams	19.05	5.65	26.15	7.64	7.10	(5.89–8.31)	1.06	Vicams vs control	8.20	(3.13–13.27)	1.23
	Control	17.85	5.55	17.95	5.57	0.10	(-0.20-0.40)	NS	Control vs ballistic	1.05	(-4.02-6.12)	NS
AROM Right Knee Extension (degrees)	Ballistic	78.15	4.77	78.25	4.93	0.10	(-0.47-0.67)	NS	Ballistic vs Vicams	4.60	(0.88–8.32)	0.97
	Vicams	78.75	4.85	82.85	4.58	4.10	(3.20–5.00)	0.87	Vicams vs control	4.95	(1.23–8.67)	1.06
	Control	77.90	4.84	77.90	4.78	0.00	(-0.40-0.40)	NS	Control vs ballistic	0.35	(-3-37-4.07)	NS
AROM Left Knee Extension (degrees)	Ballistic	77.95	3.59	78.40	3.22	0.45	(-0.02-0.92)	0.13	Ballistic vs Vicams	5.25	(2.16–8.34)	1.69
	Vicams	78.80	3.00	83.65	2.98	4.85	(3.89–5.81	1.62	Vicams vs control	6.25	(3.16–9.34)	1.46
	Control	77.35	5.30	77.40	5.29	0.05	(-0.34-0.44)	NS	Control vs ballistic	1.00	(-2.09-4.09)	NS
MVIF Right Hip Extension (N)	Ballistic	182.60	30.62	186.28	31.04	3.68	(1.77–5.59)	0.12	Ballistic vs Vicams	82.70	(55.35–110.04)	2.28
	Vicams	183.48	27.50	268.97	40.89	85.49	(77.63–93.35)	2.45	Vicams vs control	83.09	(55.74–110.43)	2.25
	Control	184.90	31.83	185.88	32.44	0.98	(-0.53-2.49)	NS	Control vs ballistic	0.39	(-26.95-27.74)	NS
MVIF Left Hip Extension (N)	Ballistic	193.63	36.25	197.30	37.40	3.68	(1.58–5.78)	0.10	Ballistic vs Vicams	89.51	(60.38–118.64)	2.21
	Vicams	193.63	28.43	286.81	43.24	93.19	(85-68-100.70)	2.55	Vicams vs control	95.64	(66.50–124.77)	2.56
	Control	189.80	30.31	191.18	30.28	1.37	(-0.09-2.83)	NS	Control vs ballistic	6.13	(-23.01-35.26)	NS
MVIF Right Knee Extension (N)	Ballistic	266.08	35.20	271.86	33.86	5.78	(0.88–10.69)	0.17	Ballistic vs Vicams	128.19	(95.29–161.09)	2.91
	Vicams	273.73	33.98	400.05	52.28	126.32	(117.19–135.45)	2.87	Vicams vs control	124.22	(91.32–157.12)	2.71
	Control	273.92	36.41	275.83	38.18	1.91	(0.08–3.74)	NS	Control vs ballistic	−3.97	(-36.87-28.93)	NS
MVIF Left Knee Extension (N)	Ballistic	267.26	49.62	270.49	48.82	3.24	(1.30–5.17)	0.07	Ballistic vs Vicams	125.83	(83.11–168.56)	2.35
	Vicams	265.00	39.78	396.32	57.90	131.32	(122.16–140.48)	2.64	Vicams vs control	124.80	(82.08–167.53)	2.17
	Control	269.61	55.42	271.52	57.14	1.91	(0.31–3.51)	NS	Control vs ballistic	−1.03	(-43.76-41.70)	NS
CMJ (m)	Ballistic	0.17	0.04	0.18	0.04	0.01	(0.01–0.01)	0.25	Ballistic vs Vicams	0.01	(-0.03-0.05)	NS
	Vicams	0.17	0.06	0.19	0.07	0.03	(0.02–0.03)	0.31	Vicams vs control	0.03	(-0.01-0.07)	NS
	Control	0.16	0.04	0.16	0.04	0.00	(0.00–0.00)	NS	Control vs ballistic	0.02	(-0.02-0.06)	NS

AROM, active range of motion; CI, confidence interval; CMJ, countermovement jump; Vicams, voluntary isometric contraction at maximal shortening; ES, effect size; MVIF, maximal voluntary isometric force; SD, standard deviation.

### 3.1 Active range of motion (AROM)

**Fig. 3:**
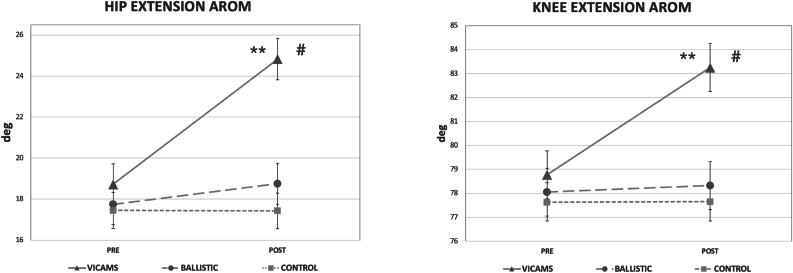
Comparison of the AROM of both hips and both knees for each group before (Pre) and after the intervention (Post). ∗Shows significant differences between p = 0.001 and p = 0.05, and ∗∗shows significant differences p < 0.001 with respect to Pre values. #Shows significant differences between p = 0.001 and p = 0.05, and ##shows significant differences p < 0.001 between groups. Data are represented as mean and SD.

In the extension of both hips, intra-group differences were observed in the VICAMS group, with higher values than baseline values (p < 0.001). In the BS group, the values also increased compared to baseline, both in right hip extension (p = 0.003) and left hip extension (p < 0.001). Between-group differences were observed in the VICAMS group after the intervention, with statistically significantly higher AROM values in the right hip extension compared to the BS (p = 0.028) and CG (p = 0.002) groups, and higher AROM values in the left hip extension compared to the BS (p = 0.003) and CG (p < 0.001) groups (Fig. 3).

Regarding the AROM of knee extension, intra-group differences were observed in the VICAMS group, as values increased compared with baseline values (p < 0.001). In the BS group, significantly higher values were observed only in left knee extension (p = 0.029). Differences between groups showed that, after the intervention, the VICAMS group obtained significantly higher values in the right knee extension compared to the BS group (p = 0.01) and the CG (0.005), and AROM values in the left knee compared to the BS and CG (p < 0.001) (Fig. 3).

### 3.2 Maximal voluntary isometric force (MVIF)

**Fig. 4:**
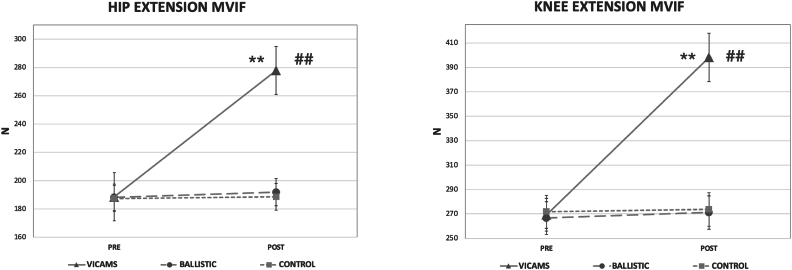
Comparison of the MVIF of both hips and both knees for each group before (Pre) and after the intervention (Post). ∗Shows significant differences between p = 0.001 and p = 0.05, and ∗∗shows significant differences p < 0.001 with respect to Pre values. #Shows significant differences between p = 0.001 and p = 0.05, and ##shows significant differences p < 0.001 between groups. Data are represented as mean and SD.

Regarding hip extension MVIF, intra-group differences were observed with higher values compared to baseline values in both the VICAMS and BS groups (p < 0.001). Between-group differences showed higher values for the VICAMS group in both hip extensions compared to the BS and CG groups (p < 0.001) (Fig. 4).

Regarding knee MVIF, intra-group differences were observed in both for the VICAMS group, with higher values compared to the baseline values (p < 0.001). In addition, in the BS group, higher values were observed in both the right (p = 0.012) and left (p < 0.001) knee than the baseline values. Between-group differences were found in the VICAMS group after the intervention, with significantly higher values in both knees compared to the BS and CG groups (p < 0.001) (Fig. 4).

### 3.3 Countermovement jump (CMJ)

**Fig. 5:**
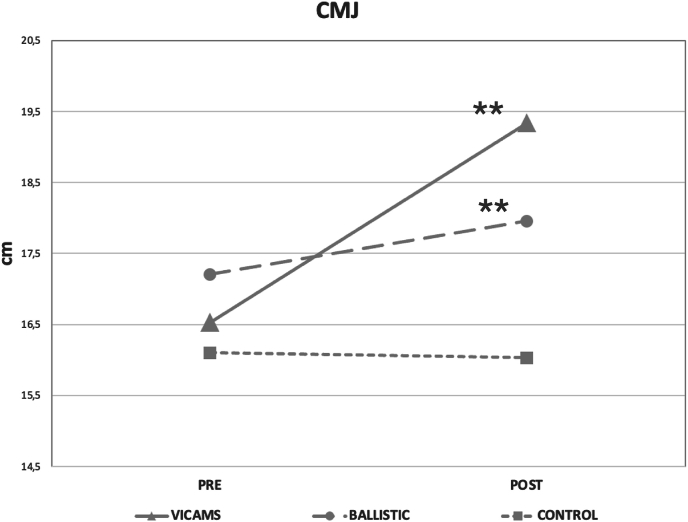
Comparison of CMJ height for each group before (Pre) and after the intervention (Post). ∗Shows significant differences between p = 0.001 and p = 0.05, and ∗∗shows significant differences p < 0.001 with respect to Pre values. #Shows significant differences between p = 0.001 and p = 0.05, and ##shows significant differences p < 0.001 between groups. Data are represented as mean and SD.

For the CMJ, intra-group differences showed that both the VICAMS (p < 0.001) and BS (p < 0.001) groups had improved values compared to baseline values. No differences between the groups were found for CMJ (p > 0.05) (Fig. 5).

## 4 Discussion

The main objective of this study was to test whether the application of VICAMS as an NR technique could affect the development of physical fitness in physically active individuals after eight weeks of training. For this purpose, it was compared with BS, and the variables AROM, MVIF, and CMJ were assessed. These data support the main hypothesis; although both groups showed improvements in all variables, VICAMS was significantly superior to BS. These results demonstrate the efficacy of VICAMS as an NR technique for improving mobility, strength, and athletic performance in healthy subjects.

In the present study, chronic improvements in AROM were observed in both the BS (2.59 %) and VICAMS groups (19.15 %). Other studies have also found improvements in flexibility after six weeks of BS application.^26^^,^^45^^,^^46^ Mahieu et al. (2007) studied Achilles tendon stiffness after six weeks of BS application and found a significant decrease in stiffness, which may have facilitated the improvement in flexibility. Chronic improvements in AROM are also attributed to the subject's increased tolerance to stretch tension.^47^^,^^48^ In contrast, BS is known to facilitate the stretch reflex and activation of the stretching muscle, which is disadvantageous for improving range of motion.^45^^,^^49^ This could justify the fact, that in our study, the improvements in AROM were inferior to those of VICAMS. As for the investigation of the chronic effects of VICAMS on AROM, the fact that flexibility is not a specific target of isometric training means that there are few studies on this subject. Hartley-O'Brien,^50^ through leg swings, and later Hardy,^51^ using passive maneuvers, managed to reach maximal muscle shortening and maintain it isometrically for 6 s. After three weeks of training, they found significant improvements of more than 15° in the AROM. Similar characteristics were established in our study, in which the maximal shortening position was actively maintained for 5 s, although in our case, the resistance was greater when external forces were applied. Subsequently, Bandy et al.^52^ actively reached the maximum range of knee joint shortening and maintained it for 5 s. They applied it for six weeks and obtained improvements in flexibility over the control group. The improvements in AROM after the application of VICAMS are justified because it is known that the use of isometric contractions of the agonist muscles and the consequent decrease in tension of the antagonist by reciprocal inhibition,^52^^,^^53^ is a good way to improve AROM.^50^^,^^51^ Kabat,^2^ stated this in the middle of the 20th century, in the first publication on the principles of NR. Furthermore, he stated the importance of voluntarily contracting the muscle from a shortened position, against the manual resistance of the researcher, to improve AROM. Based on the above, it can be suggested that VICAMS could be an effective method to improve AROM.

Our results indicated chronic improvements in MVIF development in both the BS (1.84 %) and VICAMS groups (47.63 %). Few studies have investigated the chronic effects of BS on strength performance.^54^ Cuevas-Aburto et al.,^55^ found improvements in bench press performance after four weeks of ballistic training. In contrast, after eight weeks of jump squat training, Winchester et al.^56^ found no significant improvements in maximal force production, although they did find improvements in the speed of force development. They justified these improvements in the changes in muscle fiber types. Although not a variable measured in our study, these changes in muscle fiber types could account for the improvements after BS application. Most studies on the effects of BS application on strength have focused on its immediate effects. These improvements have been attributed to increased neuromuscular activity,^57^ post-activation potentiation,^19^ and increased temperature.^18^ However, these adaptations cannot be justified, as they are responsible for long-term improvements. Regarding the effects of VICAMS application on MVIF, several investigations have linked strength development and the application of isometric training at short muscle lengths, although not at maximal shortening. Bogdanis et al.^10^ found improvements in isometric strength after six weeks of isometric squat training at short joint angles (35°), but only at angles close to the trained angle. Although muscle strength tends to increase more at or around isometrically trained angles,^58^ strength improvements have been found at other angles than the trained one.^59^^,^^60^ In line with this, Bandy et al.^14^ found strength improvements at angles other than those trained following an eight-week isometric knee extension training with 30° of flexion. These findings are consistent with our study, in which training was performed at an angle of maximal muscle shortening, and strength improved at an angle other than the trained angle. Regarding the mechanisms of improvement, changes in force production after isometric training at short muscle lengths are mainly due to neural adaptation.^12^ Kitai and Sale^61^ attributed these neural mechanisms to improvements in strength after six weeks of isometric training. Other authors have suggested that this increased neural activation occurs through spinal mechanisms to compensate for the mechanical disadvantage due to fiber overlap and fewer cross-bridges.^11^ On the other hand, the learning factor is fundamental to the chronic effects of isometric strength performance because it depends not only on the number of muscles involved but also on the ability of the nervous system to activate them adequately.^8^^,^^62^ This basic principle of NR^1^ was reaffirmed by the theory of De Luca et al.,^63^ who suggested that strength training allows the subject to learn to activate a reserve of motor units that are not normally used during maximal contractions. Furthermore, in the application of voluntary force, the major muscles must be fully activated and synergists and antagonists must be adequately activated.^8^ Carolan and Cafarelli^53^ suggested that the participants in their study learned to reduce the level of antagonist muscle activation after eight weeks of isometric training, as they gained strength despite decreased electromyographic activity of the agonist muscles. This learning in the regulation of muscle coactivation may explain the significant improvements in MVIF obtained in our study after the application of VICAMS. Based on the above, it can be suggested that the application of VICAMS may be an effective method for chronically increasing muscle strength.

In this study, there were improvements in CMJ after the intervention with BS (4.34 %) and with VICAMS (16.56 %), although there were no significant differences between the groups. Other authors have investigated the chronic effects of BS, although in a different manner than the methodology used in our study. Woolstenhulme et al.^26^ combined them with basketball practice for six weeks without finding chronic improvements in jump height. In addition, increases in jumping power have been found after eight weeks of ballistic squat training with jumping at 26–48 % of 1RM.^56^ Other studies have established that 30 % of 1RM is the most effective for improving jumping power after eight^64^ and ten weeks of ballistic jump squat training.^65^ In our study, simpler ballistic movements were used, but it appears that these improvements could be accounted for through the same adaptive processes. Although the mechanisms by which chronic effects occur following ballistic training are unclear, it is suggested that they may be produced by small changes in fiber types, changes related to recruitment issues, velocity encoding, and other neural modifications.^56^ In the case of VICAMS, several studies support the results obtained in the present study by finding improvements in CMJ height after six weeks of application of maximal voluntary isometric contractions.^10^^,^^66^, ^67^, ^68^ Bogdanis et al.^10^ reported an average increase of 7.8 % on average after the application of maximal isometric leg press contractions at short muscle lengths (35°). In contrast, another study has determined that isometric contractions did not produce increases in CMJ height.^69^ It has been suggested that this difference in results may be due to the use of single-joint exercises in a single joint position.^68^ Bogdanis et al.^10^ used a multi-joint exercise, coinciding with the present study, in which isometric contractions were applied to several joints. Bimson et al.^66^ only applied it to the knee extensors at seven different angles, including short muscle lengths, as in the present study. The improvements in CMJ were justified by the increase in hamstring stiffness and speed of force development during isometric training.^66^^,^^67^ However, isometric strength work is known to positively affect dynamic performance.^9^ Based on the results, it can be concluded that VICAMS can be an effective strategy for improving the performance of explosive actions, such as vertical jumps.

It should be noted that this study is a pioneer in the study of chronic effects of NR through VICAMS in healthy individuals. Previous studies by this research group analyzed the acute effects of VICAMS on the lower body and found similar results.^20^ Although the measurement variables were thoroughly controlled for during the study, there were not without limitations. First, the profile of the participants did not allow the results to be extrapolated to athletes. Nevertheless, the chosen profile was interesting because it represented a large sector of today's society, with an increasing number of people engaging in recreational physical activity. In addition, it would have been interesting to perform pre-post electromyographic measurements to compare the capacity for improvement in muscle contraction. Future research should focus on the effects of NR using VICAMS as a tool to improve performance in athletes. In addition, studying differences in the effects on males and females could be interesting, because of the physiological characteristics of each sex in flexibility and strength production. Finally, the resistance was applied by another person. In future studies, it would be interesting to apply other types of resistance that allow for participant independence.

## 5 Conclusions

NR using VICAMS in healthy subjects may represent an alternative to conventional strength training. In this study, the variables of active flexibility, maximal strength, and jumping obtained the best results compared with traditional training methods such as BS. Given that these variables are essential in many sports disciplines, coaches should consider incorporating this novel technique into a program to improve performance. Its applications are simple, effective, and inexpensive. Therefore, our results indicate that a new way of working in physical preparation could be developed.

## Author statement

Hernández Trujillo: Conceptualization, Methodology, Investigation, Writing - Original Draft.

González-Rivera: Conceptualization, Formal analysis, Visualization, Writing - Review & Editing.

González-Hernández: Methodology, Investigation, Writing - Review & Editing, Supervision.

## Funding

This research did not receive any specific grant from funding agencies in the public, commercial, or not-for-profit sectors.

## Declaration of competing interest

The authors declare that they have no known competing financial interests or personal relationships that could have appeared to influence the work reported in this paper.
